# Does the combination of different pitches and the absence of pitch type information influence timing control during batting in baseball?

**DOI:** 10.1371/journal.pone.0230385

**Published:** 2020-03-17

**Authors:** Shuji Kidokoro, Yuji Matsuzaki, Ryota Akagi

**Affiliations:** 1 Department of Sports Research, Japan Institute of Sports Sciences, Kita-ku, Tokyo, Japan; 2 College of Systems Engineering and Science, Shibaura Institute of Technology, Saitama, Saitama, Japan; Toronto Rehabilitation Institute - UHN, CANADA

## Abstract

Baseball pitchers use various pitch types to reduce hitting accuracy, but little is understood of the practical strategy of using visuomotor skills and timing control to respond to different pitches. This study examined 1) effectiveness of pitch type combinations, and 2) relationship between the presence and absence of advance information about the next pitch and the timing error. Twenty-six high school baseball players hit a ball launched from a pitching machine in a combination of fastballs (34.3±1.3 m·s^-1^), curveballs (25.4±1.0 m·s^-1^), and slowballs (25.5±0.9 m·s^-1^). Each participant performed three conditions. (1) Continuity condition (15 trials), in which the same pitch type was thrown five times consecutively. (2) Random condition (30 trials), in which pitch type was not preliminarily conveyed to the participants. (3) Open condition (20 trials), in which the next pitch type was preliminarily conveyed to participants. Participants’ hitting movement was recorded by an optical motion capture system and force platform. We calculated timing error based on the difference between the measured impact location (ball position relative to the batter’s body at ball-bat impact) and optimal impact location. The timing error between n-th pitch type, (n–1)-th pitch, and the presence or absence of advance information about pitch type (open vs random condition) were analyzed using three-way repeated ANOVA. The results showed that the (n–1)-th pitch type did not affect the timing of impact (*p* = 0.338). In contrast, the timing errors in open conditions were fewer compared to random conditions (*p* < 0.001). These results indicate that the pitch type sequence has insignificant effects, and advance information about pitches affects the timing errors. Therefore, having two or more pitch types, reducing the fluctuation of the pitching motion, and the early trajectory of the ball between different pitches potentially lead to increase timing errors.

## Introduction

Baseball pitchers attempt a variety of strategies to inhibit the batter from hitting the ball with the sweet spot of the bat. One strategy is to use different types of pitches such as the fastball, curveball, forkball, and change-up, and the batter must respond to those various pitches. Skilled players discern the pitch type using the visual information of the ball flight of 3 m after the ball-release (first 80 ms) [1] and empirically estimate the impact location and arrival time based on the visual information until about 150 ms before the ball-bat impact [2]. Additionally, skilled players also use visual cues about pitching kinematics before the ball release to increase their hitting accuracy [3–6]. Batters are forced to play within tight time constraints for two reasons other than the ball speed. The first is visuomotor delay [7], as the batting reaction time is approximately 200 ms [8–10]. The second is the bat swing; the batter initiates forward swings 130–280 ms before the ball-impact [11–14], then continues to accelerate the bat. Ijiri, Shinya, and Nakazawa [15] reported that the strategy to improve accuracy under severe time constraints was not to shorten the travel time but, rather, to start the movement earlier. In order to perform a well-timed impact, the start time of the forward swing is considered the most important moment, because it is difficult to make a correction for the speed or trajectory of the bat with large inertia.

The elemental events in common during the swing are the weighting of the stride (front) foot, rotation of the pelvis and thorax, and the bat’s acceleration [16, 17]. Batters cannot always perform the same series of swing movements against different pitches. The batter controls the time of weight shift to their front foot in response to different types of pitches in order to regulate the timing of the impact [18, 19]. However, these studies only considered successful trials [19] and categorized successful or unsuccessful groups based on the batted ball direction (or swing and miss) [18]. Thus, there is a lack of biomechanical and visuomotor control knowledge regarding the timing control for hitting an incoming ball. By clarifying the factors that affect temporal accuracy separately from the spatial accuracy of contacting the ball with the bat’s sweet spot, it may be possible to present a concrete strategy for batters who are unskilled at responding to different pitches.

The batter may also take the risky strategy of predicting the next pitch type based on the previous pitch if the batter keeps missing the timing of the impact without being able to respond to various pitches. Alternatively, the batter’s timing control may be unintentionally affected by the previous pitch. According to simulated studies [20, 21], a fastball thrown after three consecutive slowballs reduced the temporal and spatial accuracy of the batter. However, it is not clear whether the speed difference or trajectory difference of the pitched ball affects the impact timing of the next hit. Furthermore, it is important that the batter actually hits a pitched ball in order to obtain accurate time information about the batter’s timing strategy [22]. If the relationship between the sequence of pitches and the batter’s timing control is revealed, both offensive and defensive players can build tactics supported by scientific evidence.

Taken together, the following three factors induce early or late timing in a skilled batter: (a) the influence of previous pitches remaining in the batter’s memory, (b) a delay in judgment due to being uninformed of the next pitch type in advance, and (c) a pitch executed so powerfully and skillfully that the timing cannot be adjusted even if the batter can discern the pitch type. The impact timing appears as the complicated result of the three factors above; even considering the data acquired by Gray [20, 21], the factors affecting timing have not been identified. Consequently, the purpose of this study is to verify the following three research questions: 1) the effectiveness of the combination of pitch type, 2) the relationship between the presence and absence of advance information about the next pitch and timing error, and 3) the method of timing control for different pitch types. We hypothesized that the impact timing is affected by a combination of pitches due to speed differences, and, even if the batter gets the timing of the front foot contact wrong, they can adjust the timing by starting their pelvic rotation at an appropriate time.

## Materials and methods

### Participants

Twenty-six high school baseball players (age = 17 ± 1 year, height = 170.1 ± 4.9 cm, body mass = 66.8 ± 7.1 kg, baseball competition history = 9.8 ± 1.5 years, 13 right-handed batters and 13 left-handed batters) who belonged to the Saitama High School Baseball Federation in Japan participated in the study. All of the participants were the leading (irreplaceable) players of their teams. Informed consent was provided by participants and their guardians (i.e., legal representatives) prior to the experiment. The study was conducted in accordance with the Declaration of Helsinki and approved by the Ethical Committee of the Japan Institute of Sports Sciences (approval number: 2016013).

### Experimental procedure and task conditions

After completing warm-up exercises, each participant performed a hitting trial at an experimental laboratory. An air pressure pitching machine (TOPGUN, KYOWAGIKEN Corp., Fukuoka, Japan) was placed at a distance of 17 m from home plate. The machine was set up so that the right-handed batters were thrown balls from the right-handed pitcher, and the left-handed batters from the left-handed pitcher. The pitch types were fastballs, curveballs, and slowballs. Slowballs were only slower than fastballs, and were not drop balls like change-ups. Incidentally, it has been reported that the ball speed of the change-up fell between that of fastball and curveball [23, 24]. The participant timed the rolling ball on the sloped rail as a kinematic cue until the ball launched from the pitching machine. Since it was critically different from the kinematic cue of the pitcher’s throwing form, the participants performed enough hitting practice to be timed to the ball launched from the pitching machine before the trial. Pitched ball characteristics immediately before impact (speed, horizontal angle, vertical angle) and flight duration are illustrated in Table 1. All the participants used an aluminum bat (DeMARINI WTDX_JHPVE, Wilson Sporting Goods Company, Chicago, USA; length = 83.5 cm, mass = 900 g, center of gravity = 54.2 cm from knob end) with average inertial properties, as it would enable them to swing the bat naturally.

**Table 1:** Kinematic data of pitched balls for each pitch types.

	Fastball		Curveball		Slowball	
	Mean (SD)	95% CI	Mean (SD)	95% CI	Mean (SD)	95% CI
Speed (m·s^-1^)	34.32 (1.28)	34.22–34.43	25.37 (0.97)	25.29–25.46	25.52 (0.92)	25.44–25.60
Horizontal angle (deg)	0.33 (0.90)	0.26–0.41	-1.36 (0.90)	-1.44 –(-1.28)	0.33 (0.95)	0.25–0.41
Vertical angle (deg)	4.20 (0.60)	4.15–4.25	10.21 (1.18)	10.10–10.31	6.91 (1.05)	6.82–7.00
Flight duration (ms)	462.0 (15.8)	460.7–463.4	618.2 (20.7)	616.3–620.0	607.6 (21.4)	605.8–609.4

CI = confidence interval

**Fig 1:**
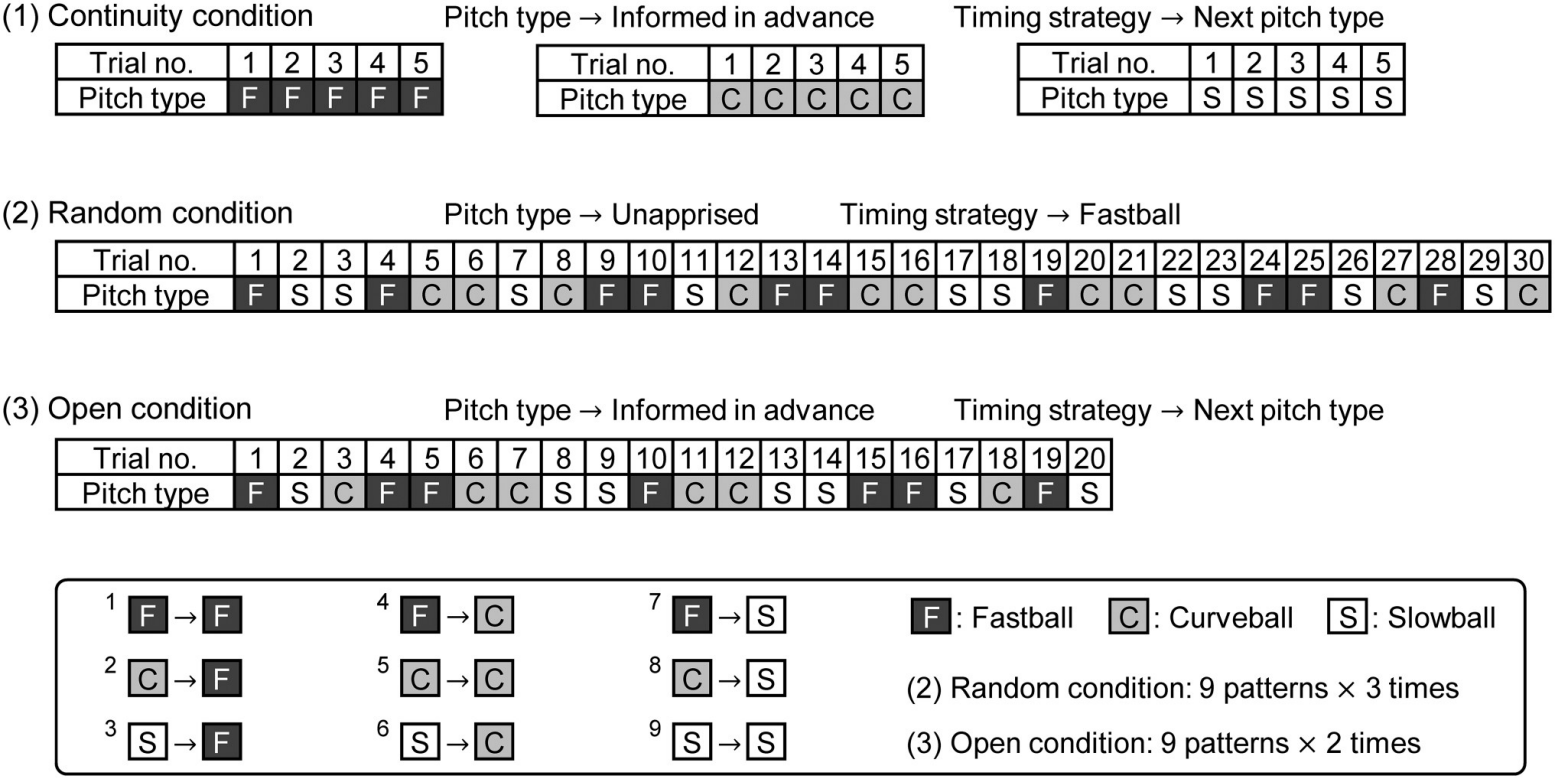
Pitch type sequence on each condition and the batter’s timing strategy for the next pitch.

Each participant performed under the following three conditions (Fig 1). (1) the continuity condition, in which they were informed of the pitch type ahead of time and the same pitch type was thrown five times consecutively; (2) the random condition, in which the pitch type was not conveyed to the participants ahead of time; and (3) the open condition, in which the next pitch type was conveyed to the participants ahead of time. In the random condition, the subjects were informed that the pitch type would be randomized, although a predetermined sequence was used. Nine different combinations were made through the combination of consecutive pairs of trials including the n-th pitch type and the previous (n–1)-th pitch type. The random condition consisted of 30 trials so that all nine combinations would collect at least three trials. In the random condition, participants were instructed not to freely predict the next pitch type but to control their timing for a fastball until ball-release. We covered the pitching machine with a sheet, except the rail over which the ball rolls to the launch position, so that the participants could not discern pitch type from the inclination of the pitching machine in random conditions. The open condition consisted of 20 trials so that nine combinations would collect at least two trials each. Based on the above method, a total of 65 trials (Continuity: 15 trials, Random: 30 trials, Open: 20 trials) were conducted with each participant. The number of trials represented an upper limit that allowed participants to maintain their concentration and prevent fatigue. The participants preliminarily received the information about the next pitch type in the trials for the open and continuity conditions, and were instructed to prepare their timing for the next pitch type. All participants underwent the experiment in a fixed sequence consisting of continuity condition, random condition, and open condition.

The batter was instructed to swing for all pitches that he judged to be strikes. Therefore, the batter had to swing even if he was thrown into a position in which he lacked skill. If the participant took a pitch because a ball was thrown outside the strike zone, we did not repeat the same trial but proceeded to the next trial in the sequence. The participants were asked to self-evaluate whether their timing of the ball-bat impact in regard to each trial was 1) early, 2) well-timed, or 3) late.

### Experimental setup

The behavior of the ball, bat, human body, and their force data during trials were recorded using a 20-camera optical motion capture system (VICON-MX, Vicon Motion Systems Ltd, UK) operating at 500 Hz and two embedded force platforms (9287B, 90×60 cm, Kistler Instruments AG, Switzerland) operating at 1000Hz. In order to measure the force from each foot independently, a separate force platform was used for each foot. A phototube sensor (E3Z-T61A, OMRON Corp., Kyoto, Japan) was placed on the pitching machine’s release point, and rectangular voltage was recorded immediately after the ball was launched. The analog signals received from the phototube sensor were input into NEXUS software used with the VICON system. Part of the data set from this study has been used in a previous study [22] which had a different research purpose.

In order to evaluate the behavior of the ball, bat, pelvis, and thorax using a motion capture system, we attached reflective markers to the participants. We also attached seven circular reflective stickers on the ball with as much distance between each sticker as possible and attached hemispherical reflective markers to the barrel and knob end of the bat. We attached spherical reflective markers to the anterior superior iliac spine (ASIS) and posterior superior iliac spine (PSIS) on the left and right sides to constitute the pelvis and attached markers to the seventh cervical vertebra and the left and right acromia to constitute the thorax.

### Data analysis

We defined the back edge of the home plate as the origin and the orthogonal coordinate system as follows: the direction from the right-handed batter’s box to the left-handed batter’s box was the x-axis, from the home plate to the center of the pitching plate was the y-axis, and the upward vertical direction was the z-axis. We reversed the positive and negative values of the x-coordinate for left-handed batters in order to treat their data as identical to those of right-handed batters.

The raw x-y-z coordinates of the bat’s markers and the body landmark markers were smoothed with a fourth-order Butterworth low-pass filter. As the bat rapidly decelerates immediately after impact, smoothing, including after impact, distorts the bat’s coordinates before impact. Therefore, we used a third-order approximate polynomial based on the data of the 20 frames before impact to extrapolate data on the 20 frames following impact; next, we smoothed them together with the raw data before impact. In order to preserve the characteristics of the raw data, the cutoff frequency was set at 35 Hz.

We obtained three-dimensional coordinates of each of the reflective stickers and estimated the center of the ball using the least-squares method. In order to quantify the timing error for each trial, we calculated the horizontal impact location (the ball’s x-y coordinates) relative to the body. The horizontal coordinates of the ball center relative to the pelvic center (calculated as the midpoint of 4 anatomical points on the pelvis) at impact was defined as the horizontal impact location. The horizontal impact location of “swing and a miss” was defined as the position at the time the ball passed through the bat’s long axis. We defined the optimal impact location based on the trials that the batters self-evaluated as “well-timed,” and calculated the timing error as the difference between the measured and estimated (optimum) y-coordinates of the impact location divided by the pitched ball speed for each trial [26]. However, since the pitch location (ball location at ball-bat impact) had been included in the variations between trials, normalization was necessary to correctly calculate the timing error for each trial. As an overall trend, the spatial ball location at ball-bat collision was located on the catcher’s side for an outside rather than an inside pitch. To take advantage of this characteristic, the information was incorporated that the optimal timing for outside pitches was later than that for inside pitches (for details, see [26]). A positive timing error indicated that the impact timing was earlier than the optimal value, while a negative timing error indicated that the impact timing was later than the optimal value. The acceptable range for the timing error differed by pitched ball speed and was ±7.9 ms for fastballs, ±10.7 ms for curveballs, and ±10.7 ms for slowballs (“Acceptable”) [26]. We divided the timing of impact into three groups (including “Acceptable”) on the basis of the impact locations; the “Early” group was classified as the ball-bat impact timed too early with the impact location positioned further on the pitcher’s side than the acceptable range, and the “Late” group was classified as the ball-bat impact timed too late with the impact location positioned farther on the catcher’s side than the acceptable range.

**Fig 2:**
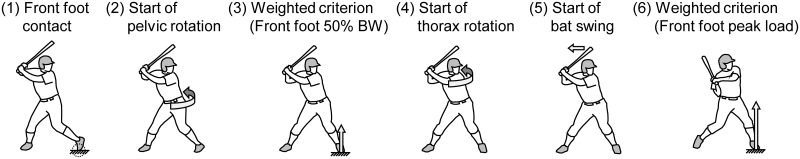
Six hitting events during a swing.

In the analytical range from ball release to ball-bat impact, we defined each hitting event as follows (Fig 2): (1) the moment the front foot (the left foot in the case of a right-handed batter) made contact with the ground, (2) the moment the pelvis started to rotate, (3) the moment the ground reaction force from the front foot exceeded 50% of the gravity acting on the participant, (4) the moment the thorax started to rotate, (5) the moment the bat started to swing forward, and (6) the moment the ground reaction force from the front foot reached its maximum. Event (3) was occasionally observed in two scenes: immediately after front foot contact and during the forward swing. If the same event was recorded twice in one trial, the latter time was adopted as one closer to the ball-bat impact. The pelvis and thorax started to rotate in (2) and (4) and this was defined as the moment of the angular velocity exceeding 100 deg/s. In addition, the bat started to swing forward in (5) and this was defined as the moment of the angular velocity of the bat’s long axis itself exceeding 300 deg/s. All the events were illustrated as negative counting to 0, which was the ball-bat impact.

### Statistical analyses

In order to verify whether the timing error was different when a series of the same type of pitches was thrown, as at batting practice, compared to when the same type of pitches occurred consecutively amid the various pitches, we conducted a two-way ANOVA (hitting condition [continuity vs. open] × pitch types [fast, curve, slow]) with repeated measures. The representative value for the continuity condition was the average of the 2nd–5th trial, and the representative value for the open condition was the average of the second trial of the same consecutive pitch types.

Additionally, in order to confirm whether the timing error was based on 1) whether the batter knew in advance about the next pitch type, 2) the n-th pitch type, and 3) the (n–1)-th pitch type, we conducted a three-way ANOVA (hitting conditions [open vs. random] × n-th pitch type [fastball, curveball, slowball] × (n–1)-th pitch type [fastball, curveball, slowball]) with repeated measures.

A two-way repeated measures ANOVA was used to assess the differences in the appearance time of each hitting event between the three timing groups (event [6 points] × timing group [fastball, acceptable vs. late; curveball and slowball, acceptable vs. early]). Here, the early group in the fastball trials and the late group in the curveball and slowball trials were eliminated from the comparison because they did not match the design for analysis.

In addition, a two-way ANOVA with repeated measures was used to assess the differences in the appearance time of each hitting event between three types of pitches in the acceptable group (event [6 points] × pitch type [fastball, curveball, slowball]). In the test of the difference in appearance time, statistical analyses were conducted for each purpose in order to clarify the standard for comparison. In the test of the difference in appearance time, statistical analyses were conducted separately instead of three-way ANOVA (6 event × 3 pitch type × 3 timing group) in order to clarify the object of important comparison.

All analyses were performed using the 24th version of SPSS (SPSS for Windows, IBM Corp., New York, USA) and statistical significance was defined as *p* < 0.05. The descriptive data were expressed as mean ±SD.

## Results

**Fig 3:**
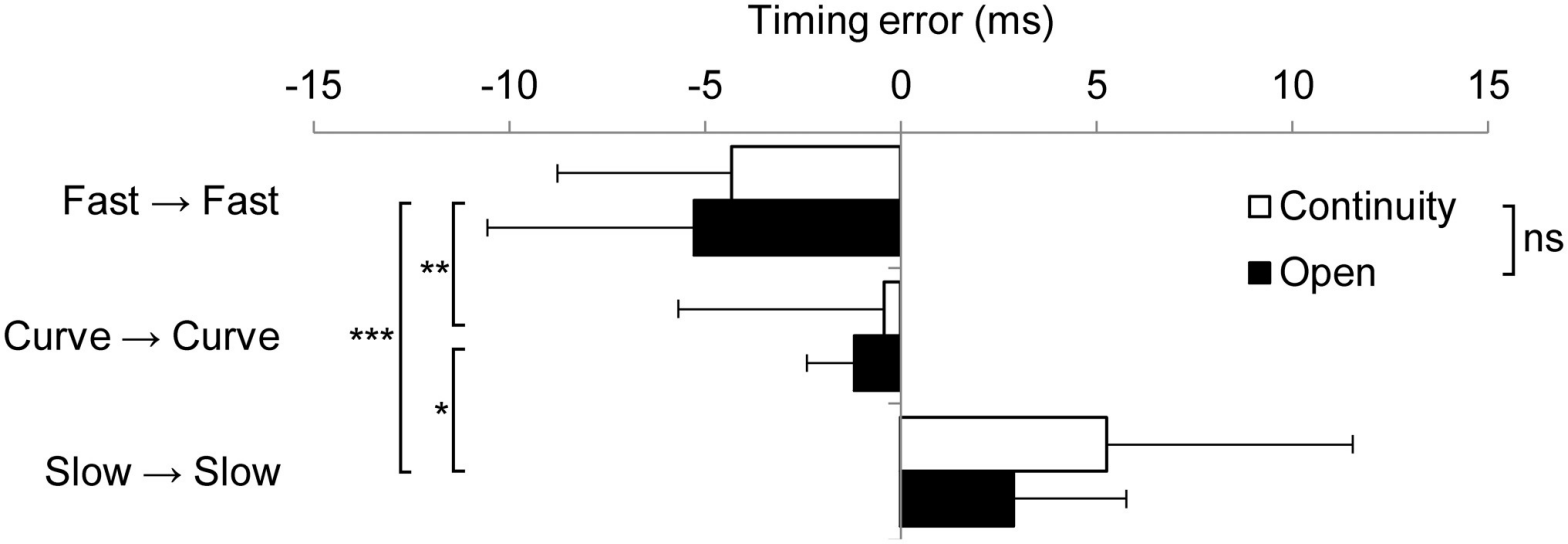
Average timing error for each pitch type compared between the continuity and open conditions. *p < 0.05, **p < 0.01, ***p < 0.001.

The two-way ANOVA demonstrated no interaction between the hitting condition (continuity vs. open) and pitch type (fastball, curveball, slowball; F(1.5, 33.4) = 0.422, *p* = 0.595), and a main effect was found in the pitch type (F(2.0, 46.0) = 18.431, *p* < 0.001). A multiple comparison found that the timing error was different for all the pitch types (fastball, –4.80 ± 4.46 ms; curveball, –0.82 ± 6.99 ms; slowball, 4.15 ± 9.40 ms; Fig 3). However, no main effect was found for the hitting condition (continuity vs. open; F(1.0, 23.0) = 2.115, *p* = 0.159).

**Fig 4:**
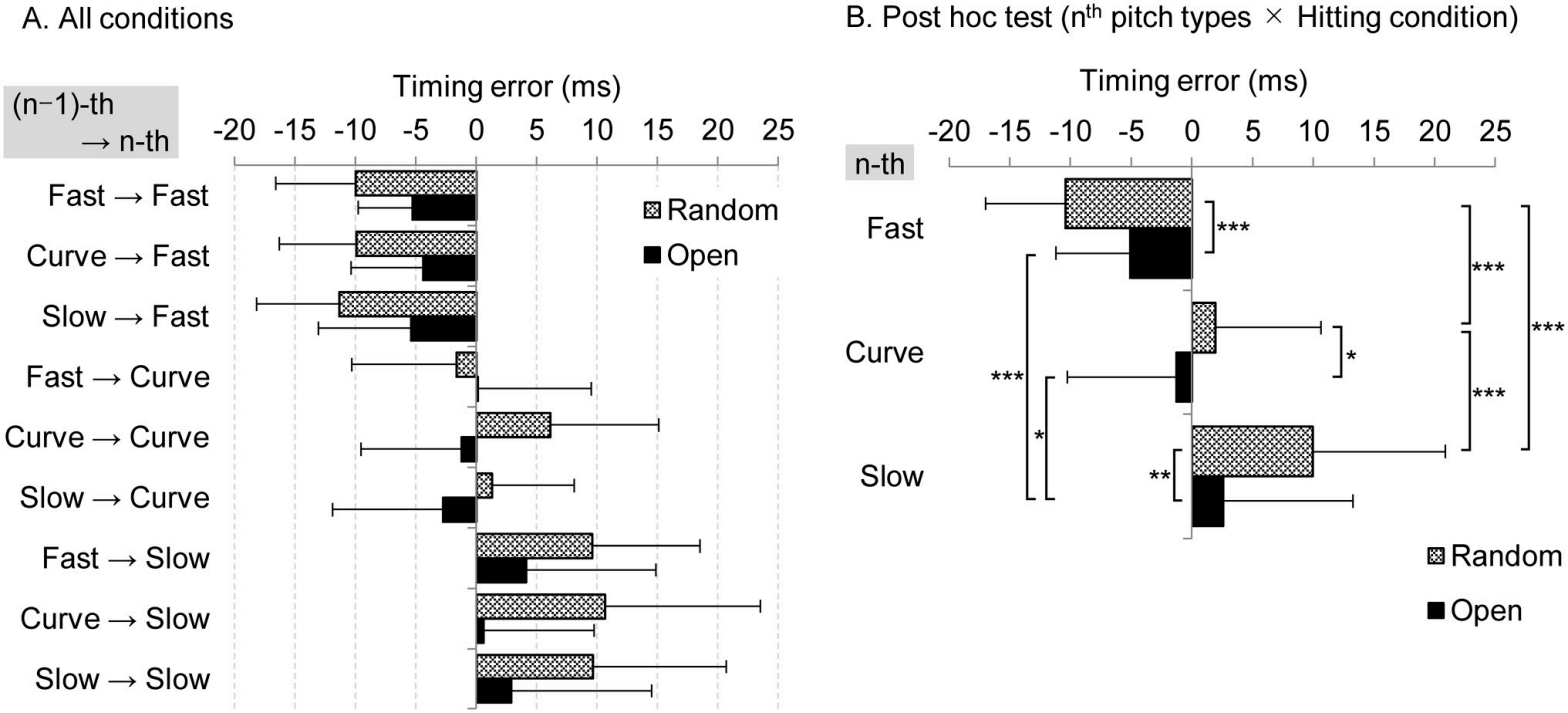
Average timing error for combinations of pitch types compared between the random and open conditions. *p < 0.05, **p < 0.01, ***p < 0.001.

The three-way ANOVA revealed that there were significant interactions between hitting condition (random vs. open) and the n-th pitch type (fastball, curveball, slowball; F(4, 88) = 19.109, *p* < 0.001). A multiple comparison of the timing error was different for the open and random condition for all n-th pitch types, and for all the pitch types except between fastballs and curveballs in the open condition (*p* < 0.05; Fig 4). There was no significant difference in the (n–1)-th pitch types (F(2, 44) = 1.133, *p* = 0.338).

**Fig 5:**
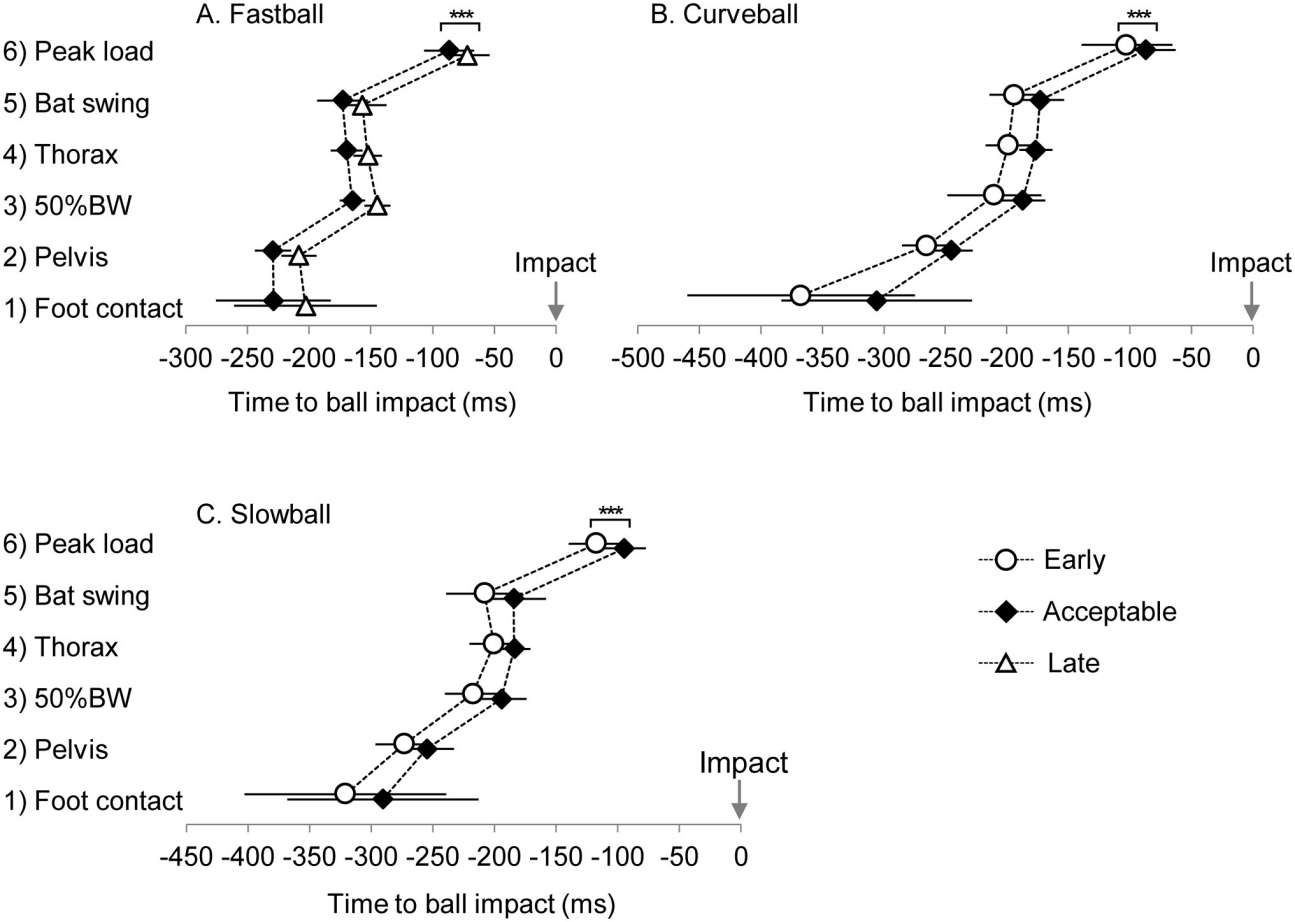
Average value of the appearance time of each hitting event for each timing group and each pitch type. ***p < 0.001.

**Fig 6:**
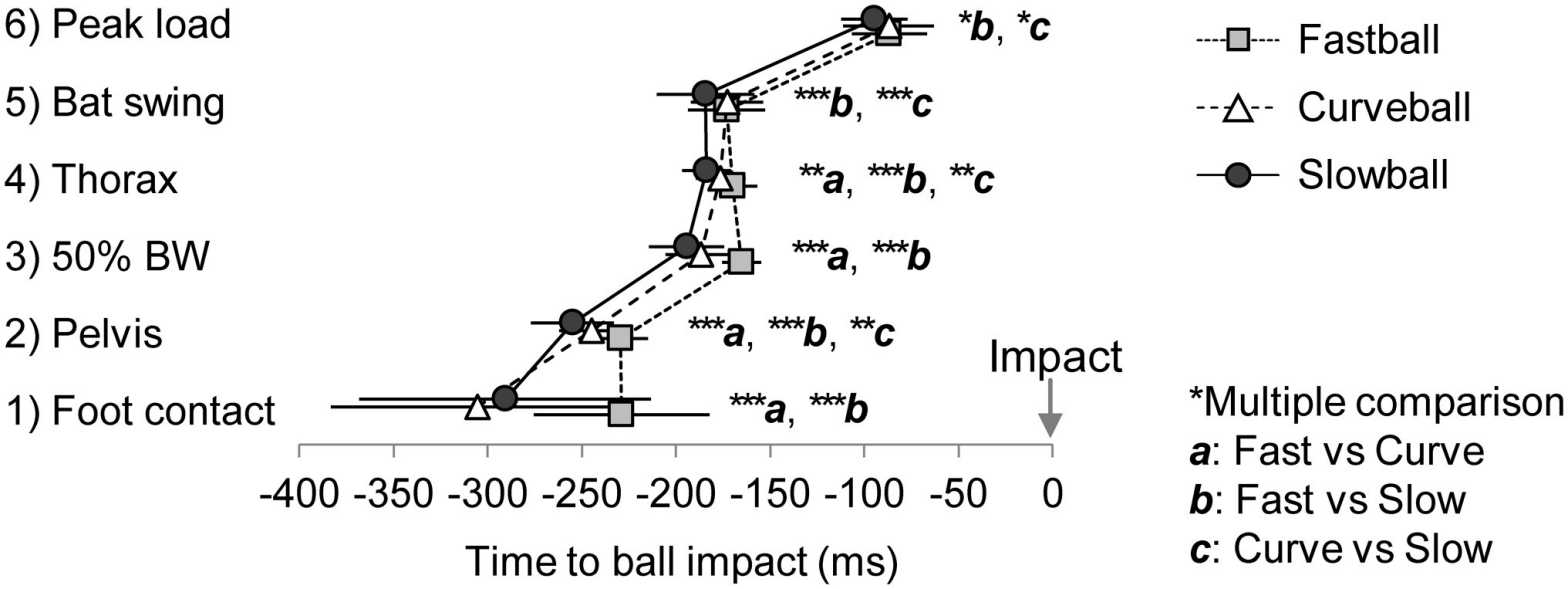
Average value of the appearance time of each hitting event for each pitch type in the acceptable group. *p < 0.05, **p < 0.01, ***p < 0.001.

The two-way ANOVA in either pitch types demonstrated no interaction between the timing groups and the appearance time of each hitting event (fastball, F(1.4, 32.9) = 2.711, *p* = 0.100; curveball, F(1.6, 35.1) = 3.270, *p* = 0.060; slowball, F(2.0, 50.1) = 1.888, *p* = 0.162), and a main effect was found in the timing groups (fastball, F(1.0, 25.0) = 171.631, *p* < 0.001; curveball, F(1.0, 25.0) = 112.93, *p* < 0.001; slowball, F(1.0, 25.0) = 63.142, *p* < 0.001) (Fig 5). In addition, an interaction was found between the pitch types and hitting event (F(3.3, 83.6) = 30.220, *p* < 0.001), and a significant difference in the appearance time for each event between the pitch types was found in all of the hitting events (Fig 6). The difference in the appearance time at the moment of front foot peak load was a maximum time of 7.85 ms between the pitch types. The standard deviation of the appearance time at the moment of front foot contact was greater than that of other events (foot contact, SD > 46.5 ms; other events, SD < 26.0 ms).

## Discussion

We investigated 1) the effectiveness of the combination of pitch types, 2) the relationship between the presence and absence of advance information about an upcoming pitch and the timing error, and 3) the method of the timing control for different pitch types. Our results indicate that timing error is not affected by previous pitches regardless of the hitting condition or types of pitches. Meanwhile, the timing error in the open condition was less than that in the random condition. In addition, the appearance time of the moment of front foot contact and subsequent hitting event in the early and late groups was different from that in the acceptable group. Even within the acceptable group, the variation in the appearance time of the front foot contact was large, and this was not only between the pitch types but also with regard to inter-individual variability within the same types of pitches.

In this study, no significant difference could be observed in the timing error between the continuity and open conditions (Fig 3). This result indicates that the temporal accuracy did not improve even under the condition of the same pitches repeating as in batting practice. In both the open and random conditions, the pitch type of the previous ball did not affect the timing error of the next trial (Fig 4). Therefore, regardless of whether the same pitch type was continued, or different pitch types were mixed, the timing error was demonstrated to depend on the ball flight cues of the next pitch type. These results are different from Gray’s findings [20, 21] and the prevailing game plan about the combination of the pitch types (pitch sequence). The absolute value of the timing error in Gray’s findings using a simulated pitcher and ball was considerably larger than that of this study. One cause of increased error in virtual simulators might be the lack of depth perception [25]. Still, a common finding can be derived from the studies even if there is a difference in the methodology with regard to actual or simulated hitting. Pitchers with more than one pitch type have an advantage in that the batter cannot focus exclusively on a single pitch type. Therefore, the pitcher must keep mixing up pitches to ensure they are not consistent with the batter’s contextual information about the pitch sequence and try to maintain the same pitching kinematics in every pitch.

The finding that the timing error differed between the random and open conditions may also be related to the manner in which the batter controls the timing for a pitched ball. Generally, hitting tasks that are more complex (e.g., visual occlusion tasks) have been found to reduce the accuracy of prediction and execution [2, 5, 14, 20, 26]. In the open condition, the batters were instructed to control their batting for the next pitch type to be thrown, while in the random condition, they were instructed to wait for a ball with the timing of a fastball. Thus, the batters delayed their preparations for fastballs compared to pitches in the open condition, because the random condition was more complicated than the open condition in that it required discrimination of the pitch type.

The appearance time in the early and late groups uniformly deviated from the time of front foot contact relative to the acceptable group (Fig 5); moreover, the timing of the front foot contact varied widely even within the acceptable group (Fig 6). Consequently, even if the batter’s front foot contact is slightly erroneous, if the batter can properly start the pelvic rotation for the next event, they will be able to correct the timing. This is similar to the findings of Katsumata [19] and Fortenbaugh, Fleisig, Onar-Thomas, and Asfour [18], who demonstrated that timing adjustments were made using a weight shift in a forward direction and the weighting of the front foot. However, front foot contact and pelvic rotation in the fastball trials occurred at nearly the same time. If a batter can execute their front foot contact so that they will not be moving too late for the fastball and if they can appropriately initiate their pelvic rotation, they will be able to respond to a wide range of pitch types in game situations.

The timing error for curveballs was less than that for slowballs pitched at the same speed (Fig 4). Previous research has also noted that early visual information is important for the batter’s pitch type recognition [1, 3]. These results suggest that the batter was able to discern the pitch type and initiate the swing motion at an appropriate moment in reaction to curveballs thrown in a more upward direction. However, if the pitcher throws a slowball into a trajectory that the batter mistakes as a fastball, the timing error might increase due to a delay in the identification of pitch type. Therefore, for many pitchers, the accuracy of ball control and consistency of pitching form between pitch types could make an equally important contribution to the pitching performance as speed properties such as ball speed and spin.

### Limitations of the study

This study has some limitations as well. We limited the objective variable to timing error and did not describe the characteristics of the batted ball. A plausible standard about timing error was established on a subjective basis for each participant [22], because there is no clear and strict definition for optimal timing.

We considered only the immediate previous pitch ((n–1)-th pitch) for the effect of pitch combinations. In addition, in the random condition, participants were instructed not to predict the next pitch type but to prepare for a fastball until ball-release to control a part of the disturbance factor. Based on the above, further studies are needed to evaluate the temporal and spatial accuracy with pitch sequence and psychological states more similar to an actual game situation.

Moreover, since the batter controls timing based on visual information including the pitcher’s body [3–6, 27], the use of a ball projection machine is a major limitation in the batting task. The acceptable timing error for ball-bat impact is about ±10 ms [22] and it is required to be of higher sensitivity. Therefore, using a ball projection machine can be an advantage partially because the batter can adjust their swing practically against the thrown ball. However, it is necessary to improve the experimental method to evaluate the timing control in an environment similar to that of a game situation, such as synchronized a ball thrown by a pitching machine with a pitcher’s motion projected onto a video screen.

## Conclusions

We demonstrated that the previous pitch type did not affect the timing of the impact. However, advance information about the next pitch type decreased the timing error. Thus, it is important for pitchers to pitch so that batters are unable to limit the next pitch type to increase the potential for developing the appropriate timing for the swing initiation. There are some specific strategies that pitchers can use to induce the batter’s false recognition; (1) having two or more pitch types and (2) reducing the fluctuation of the pitching motion and early trajectory of the ball between different pitches. Regarding the batter’s adjustment to the pitched ball, the timing deviation began from the front foot contact in the trial of the early or late impact, but even if the timing of the front foot contact was erroneous, it was possible to correct the timing by starting the pelvic rotation at an appropriate time.

## Supporting information

S1 Table The timing error and the appearance time for each condition. This is the file containing the data used for the statistical analyses in this paper.
(XLSX)
